# The International Human Genome Project

**DOI:** 10.1093/hmg/ddab198

**Published:** 2021-07-15

**Authors:** Ewan Birney

**Affiliations:** European Molecular Biology Laboratory, European Bioinformatics Institute, Wellcome Genome Campus, Hinxton, UK

## Abstract

The human genome project was conceived and executed as an international project, due to both pragmatic and principled reasons. This internationality has served the project well, with the resulting human genome being freely available for all researchers in all countries. Over time the reference human genome will likely have to evolve to a graph genome, and tap into more diverse sequences worldwide. A similar international mindset underpins data analysis for the interpretation of the human genome from basic to clinical research.

## Review

The Human Genome Project was conceived as an international endeavor (1). Partly this was pragmatism. In the mid 1980s, the scale of sequencing any organism’s genome beyond the smallest of viruses was daunting, and the human genome was almost beyond conception. A number of early ‘demonstration’ genomes, such as the Nematode was conceived as international projects, showing how such collaboration can work in practice (2). The international collaboration helped bind the academic community together, with the sense that the breakthroughs in technology and understanding were best shared, if only to ensure that you could make the soundest argument to funders at home. But it was also a matter of principle by the participants; if the human genome was going to be a key data resource for humanity, ideally a diverse group of humans should participate in its creation, and have collective ownership of the result.

Later on in the project the internationality narrowed to predominantly US and UK academic groups racing with the US company Celera to complete a draft of the human genome. This narrative often fails to bring in the Japanese and German chromosome 21, Japanese contribution to chromosome 22 and the French chromosome 14—the international community had made use of the necessity of having a clone-based map first to allow coordinated chromosomes to be delivered by specific groups. However, the overall genetic map of the human genome (using polymorphic microsatellite markers) was created by bold insightful work from the CEPH project in France in the late 1980s and early 1990s. In the latter part of the 1990s, the announcement that Celera was aiming to create a human reference genome using just whole genome shotgun sequencing altered the strategy of the academic project and narrowed its major delivery partners to four large laboratories in the US and one in the UK, with the exception of Chromosomes 21 and 14.

Despite this narrowing later in the 1990s, a key principle of international data sharing had been established. In 1997, the majority of the human genome academic project leads met in Bermuda (3) and agreed to share sequence data via the international DNA databases (ENA/GenBank/DDBJ) within 24 h of having passed QC checks. Here, the principle of the genome being a common resource for everyone was a large driver, but this also had a strong streak of pragmatism; this ‘show your data’ provided a way to coordinate the effort across the project. The end result was the announcement of the completion of two drafts of the human genome on 26 June 2000 by Bill Clinton, in the White House with leaders of both the public and private projects present, and a video call with Tony Blair to the UK. Most importantly there was a draft human genome in the public domain for all humanity to use (4,5).

This draft was progressively improved upon over the decades, with the Genome Reference Consortium (GRC (6)) providing the definitive ‘release’ of the human genome against which other information, from gene annotation through to polymorphism is described. The new releases improve representation of the genome and increasingly model aspects of structural variation. However, there is a large amount of inertia around moving between reference versions, in particular in the clinical domain (7).

## Source of human DNA

Humans, *Homo sapiens*, are a young species. There is an increasingly complex and tangled web during the latest stage of human evolution beginning some 100 000 years ago. A variety of other hominid species co-evolved and sometimes mixed with us during that founding period, but the rapid migration and expansion of humans across the world starting some 50 000 years ago has meant that human genetics (variation in human DNA) is mainly due to the variation present in Africa at this point in time. This also means there is a relatively moderate amount of large structural variation, such as large insertions, deletions or rearrangements, compared to even our great ape cousins—let alone the chaos within some vertebrate genomes. This means that for much of our genome ‘any human’ will provide a reasonable reference representation that other human genome sequences can be described against. However, there are enough regions of structural variation, in particular in important biological regions such as the major histocompatibility complex (MHC), that the choice of reference becomes an important aspect of analysis.

In the 1980s and 1990s, the workhorse scheme for isolating DNA was to create bacterial artificial chromosomes (BACs) which could each store around 250 KB of DNA stably in bacteria. The resulting bacteria could be grown as clones each containing different single regions of the human genome in a BAC; the full collection of such clones was called ‘a library.’ The public human genome sequence is made from around 50 such libraries (including some other technologies than BAC), and BAC RP11 is the most common source of information for the human genome. We can infer that the donor for the RP11 library was African-American. As such, the public human genome includes more ancestral diversity than most people appreciated, though it is still substantially biased towards recent European ancestry by regions sequenced from the other libraries.

More recently, new long-read DNA technologies from Oxford Nanopore Technologies and Pacific BioSciences have provided a new way to sequence the human genome. In particular, these technologies can span the complex repeat structures present in a variety of locations across the human genome—the centromeres, ribosomal RNA arrays and peri-centromeric repeats had been impossible to tackle with previous technology. Recently a full ‘telomere to telomere’ assembly has been released for a single human haplotype (8). As well as this being a technical tour-de-force, it opens up the potential to characterize many human genomes, if not all, in a way which can capture the complete sequence for both maternal and paternal copies.

Handling both our understanding of existing, complete, human genomes and the representation, which might be partial, of any particular individual’s genome will have to move beyond the concept of a linear reference genome with simple edits performed against this reference. The ability to think of sets of genomes as a graph elegantly solves these problems, where any type of insertion, deletion or rearrangement from one sequence to another can be represented. ‘Graph genomes’ have been used in sequence analysis and bioinformatics since the early 2000s (9), with diverse applications spanning assembly through to splicing patterns, but their routine use for representing, annotating and manipulating information beyond these select applications has been limited. As more and more human genomes are generated in an end to end manner, and as there becomes more appreciation of the biology present in some of these more tangled regions we will have to have better tools, visualizations and mindset that can accommodate this representation. Indeed, this ‘multiple genome’ problem is present in representing each individual’s diploid haplotypes, and so is a direct concern for a complete view of an individual’s human genome (10). We should be thankful that we do not have the genomic complexity of most other metazoa with far higher levels of structural variation, let alone the complex polyploid structures present across plants.

## Internationality in both Research and Clinical Human Genetics

The human genome provides a natural ‘index’ for all the RNAs and proteins made in a cell, and has been a key part of basic research in the design of reagents (from microarrays in 2000s to CRISPR libraries in the 2020s) and the interpretation of results from RNAseq through to Mass-spec proteomics. Much of this is supported by the presence of large scale open access databases in molecular biology, which aggregate this information for all scientists to use worldwide (11,12). In addition, the human genome has revitalized human genetics—the study of human biology using natural variation present between individuals. In the latter case, the combination of cheap genotyping (still predominantly via microarrays) and cheap sequencing (via the short-read technology of Illumina) has allowed the routine generation of near complete maps of individuals for their common DNA variation (common meaning present in around 1% of individuals or more). The cheapness of this genetic assay has allowed for large scale genotyping, and increasingly now full sequencing, of cohorts to occur in many places across the world. The mainstay analysis of these cohorts is genome-wide association studies (GWAS), described in more depth in this special issue. More recently, the cheapness of effective short-read sequencing, which can capture the majority of changes in protein coding genes has shifted clinical genetics from targeted gene-by-gene diagnosis to a global whole exome (WES) or whole genome (WGS) approach.

For both research and clinical analysis, responsible international data analysis has been critically important in unlocking insights. In the former research setting, replication between cohorts was important to generate confidence in the results of GWAS. This has shifted to the almost routine global consortium around a particular phenotype to maximize power; the presence of diverse cohorts not only increases the power around each tested variant, but the differential frequencies of rarer alleles means that each cohort ‘sees’ a slightly different spectrum of variants. These mega-author list papers march on, and despite the slightly repetitive nature of the science, each phenotype under study is worth understanding in as much detail as possible. Similarly in rare disease genetics, where there might be a handful of individuals worldwide who have the same mutation, international collaboration has been key to providing robust diagnosis and gene discovery for human genetics. This has been codified in projects such as the Matchmaker exchange (13,14), which allows clinical genetics groups to exchange information of genes of interest in a secure, responsible and even-handed manner.

Like much of the developing world, African nations are now bringing in more genetics research using the technologies developed over the previous decade and are now organizing research cohorts and deploy human clinical genetics more broadly across Africa; this is the start of rebalancing inequity in this area of research in general, but is also an opportunity globally as the richest source of genetic diversity in humans is found in the continent of the birthplace of our species. More recently the excellent H3Africa (15) resources, led by African scientists, have been creating more research cohorts that span different nations in Africa. Whilst keeping the African-led nature of this project, and placing African scientists to the fore, H3Africa has also committed to responsible data sharing. Similar efforts to H3Africa and continuation of H3Africa’s work itself are needed to broaden the practice of genetics and genomics globally over the coming decades.

To enable the most utility from these datasets, we must have responsible joint data analysis of both research cohorts and secondary use of clinical genomics. Such data sharing must be rooted in the ethical framework and the legal processes derived from them present in each country. Furthermore, international data analysis necessitates international standards for the datasets. Here the Global Alliance for Genomics and Health (GA4GH) is an organization founded in 2014 to enable responsible data sharing in genomics globally. Nearly every country has the goal to better understand the health and disease present in its population via science, and this broad goal is present in the UN Charter for Human Rights (16).The GA4GH ethical frameworks aim to activate these rights and align the discussions happening in many countries for responsible global data analysis; in practice, this maps to easier mutual recognition of processes and concepts. On the technical side, the entire endeavor of human genomics, from its earliest days in the 1980s have required well understood data structures and protocols to share data or analysis, often created as de facto standards between academics by virtue of the need to share data. GA4GH provides a responsible home for these standards (such as the widely used BAM/CRAM and VCF standards) and a process for creating new standards in the Cloud-enabled and connected world we live in now.

## Conclusion

The human genome is a dataset which is owned by all of us, for use by humanity. Human genetics and genomics has always flourished in an international context and leaders of the field in the 1970s, 1980s and 1990s insisted on international, open data sharing of key resources. The future is likely to be as demanding for the need for as open as possible data sharing, adapting to the world of even more genetic and genomic data, again for the benefit of all humanity.
